# Supplementary material for: Allele imputation for the killer cell immunoglobulin-like receptor KIR3DL1/S1
Source: PLoS Comput Biol. 2022 Feb 22;18(2):e1009059. doi: 10.1371/journal.pcbi.1009059 (PMC8896733; doi:10.1371/journal.pcbi.1009059)
Supplement: Attachment — Submitted filename: KIR_Imputation_Response_to_Reviewers.pdf [file pcbi.1009059.s006.pdf]

Dear Reviewers and Editors,

Thank you for taking the time to contribute constructive feedback for our paper "*Allele imputation for the Killer cell Immunoglobulin-like Receptor KIR3DL1/S1*". Below we provide responses to each comment as well as the relevant text from the manuscript (shown in blue), where corresponding changes have been made. These changes are also highlighted in one of the submitted versions of the manuscript, as requested. Major revisions include expanding our optimization of PONG to three low density SNP arrays: the Infinium, Immunochip and Multi-Ethnic Global arrays. We conducted these analyses on populations from East Asia and Africa, thereby expanding this portion of the study from the Norwegian population that we analyzed previously. In the comments we have also provided an explanation of why PONG is currently focused on *KIR3DL1/S1,* as this point was raised by both reviewers as well as the editor. Thank you for your continued consideration for the publication of this manuscript in PLOS Computational Biology.

With Warm Regards,
Drs. Harrison and Norman

Reviewer #1

Reviewer #1: This manuscript by Harrison and colleagues describes an interesting adaptation of the widely used HIBAG method for HLA allelic inference from SNP array data, making it possible to impute allelic variation of the KIR3DL1/DS1 gene. Given the demonstrated functional differences of KIR alleles with regard to expression and affinity to their HLA ligands, as well as allele-specific disease associations, the work is an important contribution and very useful for the immunogenetics research community. In contrast to available WGS- or WES-based KIR inference methods that rely on the availability of sequence-level information, PONG allows large-scale imputation of KIR3DL1/DS1 allelic data from cohorts with available array-based SNP genotyping data.

The reported KIR typing accuracy is high, and is comparable to commonly used HLA imputation methods. As the authors discuss, accuracy is slightly lower for populations with higher allelic diversity, which is indeed not unexpected and helps to make the case for increased research efforts in these populations.
The manuscript is well written, and the rationale clearly described.
Adapting the HIBAG method allowed the authors to provide the imputation models to the scientific community without having to share individual-level genetic data, which is often not possible due to IRB regulations and ethical concerns.

Major points:
1) Either expand to other KIR genes or explain better why we aren't:
KIR3DL1/DS1 is not the only KIR gene with functionally relevant allelic diversity. Once the modified HIBAG method is established, it should be conceptually straightforward to also impute allelic diversity for other KIR genes. For example, KIR2DL3 and KIR2DL2 can have different binding affinities to their HLA-C C1 and C2 ligands depending on allele status. When reading the paper, I expected a discussion why other KIR genes were considered out of scope. Do you consider them less relevant? Are there significant issues related to the complexity of the region (copy number, etc.) that make this more difficult, and that I am not aware of (not unlikely, in which case I'd like to learn)? Are you going to tackle those next (yes, please)?
In its current form, it felt a bit like reviewing an HLA imputation method that only imputes e.g. HLA-B. Even if very accurate, it's important to consider downstream association results with disease phenotypes in the context of the haplotype structure. Since e.g. KIR3DL1 and KIR2DL3 are both present on the most common KIR A haplotypes, but in different combinations on KIR B haplotypes, inferring causality relies on the ability to correct for the presence / absence of other genes and alleles.
To be clear, I think PONG is definitely useful in its current form, and I will for sure use it myself, but I'm missing a good explanation for the narrow scope, given how much an addition of other genes would add in terms of usefulness.

Response: We chose KIR3DL1/S1 specifically because this has been explicitly and reproducibly implicated in the widest range of human disease or therapy responses for any of the KIR that have been studied. We note that in many of these cases, knowledge of KIR3DL1/S1 allotype diversity has been sufficient for understanding or diagnosing the role of KIR in those conditions. Thus, we recognized the urgent need to develop this package and make it available for other researchers and clinicians. As both reviewers have stated, we have achieved these goals and, thanks to the preprint posting, the package is currently being used by multiple groups.

The second goal of the study is to provide a proof-of-concept that alleles of highly polymorphic *KIR* genes could be imputed from whole-genome SNP data. Mainly for the reasons above, *KIR3DL1/S1* is the locus for which we have by far the greatest amount of experience and, importantly, control data and specimens. We generated and extensively validated the *KIR3DL1/S1* genotypes from the test subjects described. To

generate and validate control and model data for the remaining KIR will take a similar endeavor and in our experience each *KIR* locus can present its own challenges to being genotyped. Thus, we also recognize the need for full-KIR imputation and are working towards these goals, having received a 5-year grant for the purpose.

To address these comments, we have added a paragraph to the discussion explaining our reasoning for focusing on *KIR3DL1/S1* and why this is an important step towards ultimately imputing all *KIR* alleles.

P24 L528: Due to their established associations with multiple human ailments including infectious disease, cancer and autoimmunity [1], we focused this study on determining the allotypes of *KIR3DL1/S1*. Included among those with the greatest imputation accuracy are common allotypes having altered or no expression, specificity-determining polymorphism, and an activating variant [2]. Any of these allotypes can have dramatic consequence for the function of NK cells. Thus, knowledge of *KIR3DL1/S1* genotype, in combination with *HLA-A* and *-B* genotypes, is often sufficient to understand the disease mechanism or move the field forward in other ways [15, 52, 103, 107]. In other cases, it will be necessary to form a complete picture of KIR interactions with HLA-C in addition to -A and -B, by generating a genotype from the entire *KIR* locus [29]. Validation of PONG to impute *KIR3DL1/S1* is a robust proof of concept that can be built upon to impute entire *KIR* region genotypes. Expanding to other *KIR* genes will be challenging as the genomic region contains substantial structural variation and, unlike *HLA*, classifiers are sampled from the entire locus and flanking parts, rather than individual genes. We conducted rigorous testing to determine the efficacy and limits of HIBAG in imputing *KIR3DL1/S1* genotypes. This was a first but important step before expanding the concept in future work to impute complete *KIR* haplotypes.

Minor points:

2) Add a cancer reference for lines 43-46:

- In the introduction, the authors mention HIV research and cancer therapy as key areas impacted by KIR/HLA diversity, but the cited references 43-46 only refer to HIV. Please add some cancer-specific references.

Response: We added three references here that show how KIR3DL1/S1 specifically plays an important role in cancer immunotherapy responses. Ayello et al (2017) illustrate an increase in KIR3DL1/S1 expression in response to B-cell non-Hodgkin lymphoma and expansion of NK-cells associated with decrease in tumour burden. Expanded NK cells are proposed as an adoptive cellular immunotherapy. Makanga et al (2021) show that KIR3DL1/S1 expression can impact treatment of cancers with rituximab, a CD20 inhibitor. Finally, Trefny et al (2019) discovered a variant that is associated with resistance to PD-1 blockades, a therapy for patience with non-small cell lung cancer.

P33 L800:

47. Ayello, J., et al., *Genetically re-engineered K562 cells significantly expand and functionally activate cord blood natural killer cells: Potential for adoptive cellular immunotherapy.* Experimental hematology, 2017. **46**: p. 38-47.
48. Makanga, D.R., et al., *Low number of KIR ligands in lymphoma patients favors a good rituximab-dependent NK cell response.* OncoImmunology, 2021. **10**(1): p. 1936392.
49. Trefny, M.P., et al., *A variant of a killer cell immunoglobulin-like receptor is associated with resistance to PD-1 blockade in lung cancer.* Clinical Cancer Research, 2019. **25**(10): p. 3026-3034.

3) Asses the accuracy in other SNP arrays.
- It would be great to further assess differences in imputation accuracy from other SNP arrays with low marker density in the LRC region.

We added experiments testing and optimizing PONG against three commonly used low density SNP arrays: the Infinium, Immunochip, and Multi-ethnic Global Arrays. We found that increasing the genome window size, coupled with pre-imputation increased the number of available SNP classifiers and produced *KIR3DL1/S1* genotypes approaching the accuracy of the high-density chip we originally targeted. We added an additional set of models based on the extended window to the Github site for public use. We feel this was an extremely valuable exercise that has increased the immediate utility of our imputation package and are grateful for this suggestion. A table (Table 2) was added to illustrate the results.

P7 L153: We established that we could impute *KIR3DL1/S1* alleles using data from multiple low-density SNP genotyping platforms.

P10 L215 (Methods): ***Imputing KIR3DL1/S1 alleles from Low Density SNP Arrays***
The next goal of this study was to test the accuracy of *KIR3DL1/S1* imputation using data from commonly used low density SNP arrays. From the 1,000 Genomes sequence data vcf files [79], we extracted those chromosome 19 SNPs equivalent to the Immunochip (4,902 SNPs, 26 in *KIR* region), Infinium (23,117 SNPs, 18 in *KIR* region) or MEGA (36,534 SNPs, 76 in *KIR* region) arrays. To increase the number of available classifiers, we built a second Global model in which we expanded the target region to chr19: 55,100,000 – 55,500,000 (hg19). These coordinates match those of the KIR*IMP program, which can be used to impute *KIR* gene content genotypes [88]. We then built and tested *KIR3DL1/S1* allele imputation models as described above. We used the Michigan imputation server [89] to supplement the low-density data with Illumina Omni 2.5 array data (1,832,506 chromosome 19 SNPs, 4,093 in *KIR* region). Input data was first phased with EAGLE [90] and an RSQ filter of 0.3 was applied to ensure imputation accuracy [91].

P17 L383 (Results): ***Imputing KIR3DL1/S1 alleles using low density genotyping datasets***
We evaluated whether *KIR3DL1/S1* genotyping could be performed using data obtained from each of three commonly used low density arrays. For this test, we included the AFR and EAS groups because they represent the highest and lowest genetic diversity, respectively, of the five major population groups in the 1,000 Genomes database. We based this test on the Global model and extended the window from which classifiers are sampled to chr19: 55,100,000 – 55,500,000 (hg19). To ensure continuity, we used the same individuals as the Global model development described above. Using the SNP genotype data directly produced poor results for each of these chips (**Table 2**). Thus, we first supplemented the data by imputing Illumina Omni 2.5 array SNPs. Accuracy in the AFR population improved to 78%, 80% and 84% when the genotypes originated from Infinium, Immunochip or MEGA SNPs, respectively (**Table 2**). For the EAS group the accuracy was 81%, 89% and 92% for Infinium, Immunochip and MEGA arrays, respectively (**Table 2**). As expected, the accuracy was lower than that obtained directly from the high-density Illumina Omni 2.5 data (88% AFR and 97% EAS, **Figure 3**). That we observed little difference for the EUR group between low- and high-density arrays, with a minimum of 91% accuracy, may be due to original ascertainment bias in chip design. Consistent with this reasoning, the MEGA chip, which was designed to reduce such bias [94], gave the greatest accuracy through this test. In summary, these results show it is tractable to use PONG with low-density arrays when higher-density array genotypes are first imputed from the starting data.

4) Expand on discussion about using with KIR*IMP:
- The authors mention the possibility of using KIR*IMP in addition to improve gene presence / absence inference. I applaud the author's decision to make PONG freely available to the scientific community.

Unfortunately, KIR*IMP doesn't offer the same license model. Also, individual-level genotype e data needs to be uploaded to a server, which is often not possible due to IRB restrictions. I think the latter should be mentioned when suggesting the joint use of both methods.

Response: We added text to the discussion that states the two strategies.

P23 L491: KIR*IMP does not require installation and is run through a web interface via data upload.

P23 L500: PONG is open source and gives users the ability to publish reference models that do not contain information about individuals, nor is the upload of genotype data to any public server required. These features both support reproducibility of research and increase efficiency of the imputation as the models are expanded.

5) Explain benefits of HIBAG for our own program:

- The authors might consider putting more emphasis on the advantages of the HIBAG method, allowing researchers to **publish reference models** for imputation without the requirement to share whole panels with individual-level genetic information, which can limit the usefulness of alternative methodological approaches.

Response: We have added text to the Discussion explaining that PONG can be used offline and with other models in addition to those we made, therefore increasing both the utility of the method and the reproducibility of published results.

P23 L500: PONG is open source and gives users the ability to publish reference models that do not contain information about individuals, nor is the upload of genotype data to any public server required. These features both support reproducibility of research and increase efficiency of the imputation as the models are expanded.

Reviewer #2

Reviewer #2: The study by Harrison et al utilizes a machine-learning approach to accurately impute alleles of the highly variable KIR3DL1/S1 gene from high density SNP data. This genetic system encodes proteins on the surface of NK cells and in combination with specific ligands (HLA; another polymorphic system) have been shown to be associated with a number of immune-mediated diseases. Given the complexity of the KIR genetic region on chr 19, high-resolution KIR genotyping is typically needed to evaluate this genetic system in disease association studies. This study describes a valuable computational tool to impute KIR3DL1/S1 genotypes based on genome-wide SNP data that is often available in genetic-based association studies. Furthermore, the accuracy of the tool (PONG) is comparable to levels obtained from other programs to input HLA.

There are certain aspects that the authors should clarify regarding the analysis as indicated below:

i) In the methods section on page 8, it should be clear that the KIR genotyping determined by PING is based on sequence data. It is unclear from the text that this is the case and requires examination of reference 77. Furthermore, it is unclear what is the relevance of the 143 subjects with Sanger-based sequencing. If this was also used for KIR genotyping then this raises issues related to this approach including phasing.

Response: We clarified the Methods and other sections to say that we used NGS sequence data to generate the *KIR3DL1/S1* genotypes. We removed the sentence regarding the Sanger sequencing of the cohort of 143 individuals -these were 1000 Genomes individuals from whom we had both NGS and Sanger sequence data, and we agree this information was not relevant to the present study.

P7 L154: We optimized the process using 1,000 Genomes individuals, because we had previously determined their *KIR3DL1/S1* alleles from sequence data [80] and high-density SNP data (Illumina Omni 2.5 chip) is available from this cohort [79].

P8 L163: To determine the *KIR3DL1/S1* alleles present in each individual we used the Pushing Immunogenetics to the Next Generation (PING) pipeline on high-depth sequence data, as previously described [80].

P12 L256: We used SNP data from the 1,000 Genomes project [79] and KIR3DL1/S1 genotypes that we had previously determined from sequence data from the same individuals [80].

P27 L591: The alleles were determined from short read sequence data [80].

ii) The use of a second SNP platform with a set of Norwegian subjects is useful to determine the value of PONG with a SNP array with a different density across the relevant region. However, could the outcome be an over-estimate given the likely higher linkage disequilibrium and reduced genetic diversity in this population relative to others such as an African cohort?

To address this question and one from Reviewer 1, we expanded the analysis to test the possibilities of running PONG using multiple low-density arrays and included the AFR cohort in those tests. The approach and the results are detailed above in the response R1.3 (methods P10 L215 and main text P17 L383). In summary, we agree there will likely remain a greater accuracy using data from Europeans,

potentially due to SNP chip bias as well as the increased LD/lower diversity compared to Africans, and now we have tested and shown the differences across several platforms.

iii) There appears to be a discrepancy in the sensitivity values in the abstract and author's summary.

Response: Thank you for catching this mistake. We have corrected the reported specificity value for the global model in both in the Abstract and the Summary section.

Abstract, P2 L38: Our Global imputation model is effective at genotyping *KIR3DL1/S1* alleles with an accuracy ranging from 88% in Africans to 97% in East Asians, with mean specificity of 99% and sensitivity of 95% for alleles >1% frequency.

Author Summary P3 L63: Globally, imputation of *KIR3DL1/S1* alleles having frequency >1% has a mean sensitivity of 95% and specificity of 99%.

Results P16 L344: The mean specificity across the 42 alleles was 99% with only two alleles having a specificity below 99% (**Figure 3C**).

Results P16 L356: Conversely, *KIR3DL1/S1* alleles with a frequency above 1% had a mean sensitivity of 95%.

iv) The lower accuracy in the African subjects (89%) is as expected given the lower LD and diversity but can the authors explain the lower % for the South Asian subjects? Is this different to what is observed for the HLA imputation tools?

Response: The lower accuracy of *KIR3DL1/S1* imputation in the South Asian group is likely due to several factors: Both *KIR* allele and genome-wide SNP diversity of South Asians remains under-characterized, we observed a high number of low-frequency alleles in the South Asian group we studied, and we previously observed a higher frequency of *KIR* locus structural variants in this population. Any of these factors could reduce the accuracy of imputation. We added the following three parts to the text to discuss these reasons:

P17 L373: The AFR, AMR and SAS population groups had the highest number of KIR3DL1/S1 alleles of frequency less than 1% in the test set (16, 10 and 10, respectively). Contributing to the lower accuracy of PONG in AFR and SAS, these low-frequency KIR alleles accounted for over 50% of those present in each group. By contrast, only three such low frequency alleles were present in the EAS group. In summary, the accuracy of PONG is affected by the frequency of KIR3DL1/S1 alleles and is therefore less effective in more diverse human populations given a similar-sized training sample. Because we have filtered on minor allele count, the imputation accuracy in these populations will increase with the size of the model data set used.

P22 L472: As observed for *HLA* [104], we found a negative correlation between the accuracy of PONG and the diversity of *KIR3DL1/S1* alleles in a population. For example, the African population group we studied has the highest number of distinct *KIR3DL1/S1* alleles as well as the highest number with a frequency below 1%. We also observed a high incidence of alleles at less than 1% in the South Asian group, as well as a higher frequency of haplotypes having duplicated *KIR3DL1/S1* compared with Africans [93]. The result is that imputation accuracy is lowest in Africans and South Asians.

P22 L478: Finally, KIR3DL1/S1 allelic diversity is under-characterized for many non-European origin populations. Any KIR3DL1/S1 alleles newly identified in these populations will need to be incorporated into future models to increase imputation accuracy

HIBAG was not originally tested in South Asian populations, but subsequent studies have tested the efficacy of HIBAG in a population from India. HIBAG performs slightly better for imputing HLA alleles in the Indian population tested (94% accuracy), with the exception of HLA-B. HLA-B alleles were imputed with an accuracy in Indians at 88% [3] which is similar to the accuracy we were able to achieve for KIR3DL1/S1 imputation.

P24 L517: Whereas HIBAG did not originally include South Asian populations, subsequent studies in an population from India found an imputation accuracy of 88% for *HLA-B* rising to 94% for other *HLA* genes [91].

v) Can the authors justify the use of the EUR subjects to determine the threshold/filtering parameters?

Response: A major goal of our work is to facilitate health research for currently under-represented population groups and to assist this aim, the ultimate objective of this study was to develop a multi-ethnic imputation platform for KIR3DL1/S1. Nevertheless, the European population remains the most extensively characterized for immunogenetic as well as genome diversity and, coupled with having the fewest gross number of *KIR3DL1/S1* alleles of the groups studied, provided the greatest confidence for developing the algorithm. In response to this comment, we went back and tested the other populations and have now shown that the parameters we optimized in Europeans produce the greatest accuracy in each of the other populations as well. We now include these results as a Supplemental Table (S4).

P9 L196: We chose the EUR subset to develop the filtering thresholds because this was the group having the fewest number of *KIR3DL1/S1* alleles before filtering, and because European groups are currently the most extensively characterized [84-87].

P13 L288: Moreover, these parameters produced the greatest imputation accuracy across all five major population groups (**Table S4**). We therefore implemented these parameters (MAC < 3 both for SNPs and *KIR3DL1/S1* alleles) in all subsequent analyses.

P 29 L 629: Table S4 (xlsx) Filtering parameter optimization for Imputation Models

vi) 3DL1/S1 is an important gene in the KIR complex but there are a number of other KIR genes in the region that also bind Bw4 and the C1/C2 ligands and are variable. Have the authors attempted to impute the genotypes of these other genes?

Response: This comment is similar to that raised by Reviewer 1 and we draw attention to the response to R1.1, and the section of text that we added. Briefly, due to the interaction with Bw4+ HLA-A and -B in multiple health scenarios, there was an urgent need to provide a means for imputing *KIR3DL1/S1* alleles from genome data (we should note that KIR3DL1/S1 is the only KIR that binds Bw4+ HLA as far as we are aware). Secondly this study will serve as a proof of concept that the complex task of imputing all the *KIR* alleles could ultimately be accomplished.

P24 L528: Due to their established associations with multiple human ailments including infectious disease, cancer and autoimmunity [1], we focused this study on determining the allotypes of *KIR3DL1/S1*. Included among those with the greatest imputation accuracy are common allotypes having altered or no expression, specificity-determining polymorphism, and an activating variant [2]. Any of these allotypes can have dramatic consequence for the function of NK cells. Thus, knowledge of *KIR3DL1/S1* genotype, in combination with *HLA-A* and *-B* genotypes, is often sufficient to understand the disease mechanism or move the field forward in other ways [15, 52, 103, 107]. In other cases, it will be necessary to form a complete picture of KIR interactions with HLA-C in addition to -A and -B, by generating a genotype from the entire *KIR* locus [29]. Validation of PONG to impute *KIR3DL1/S1* is a robust proof of concept that can be built upon to impute entire *KIR* region genotypes. Expanding to other *KIR* genes will be challenging as the genomic region contains substantial structural variation and, unlike *HLA*, classifiers are sampled from the entire locus and flanking parts, rather than individual genes. We conducted rigorous testing to determine the efficacy and limits of HIBAG in imputing *KIR3DL1/S1* genotypes. This was a first but important step before expanding the concept in future work to impute complete *KIR* haplotypes.
